# Supplementary material for: Co-transcriptional splicing changes combine with reduced productive transcription initiation for cold-induced repression of FLC
Source: Nucleic Acids Res. 2026 Jan 8;54(1):gkaf1462. doi: 10.1093/nar/gkaf1462 (PMC12781886; doi:10.1093/nar/gkaf1462)
Supplement: gkaf1462_Supplemental_Files [file gkaf1462_supplemental_files.zip › maple_et_al_supplementary_information_17102025.pdf]

Supplementary Information

Co-transcriptional splicing changes combine with reduced productive transcription initiation for cold-induced repression of *FLC*

Robert Maple[1,†], Govind Menon[2,†], Susan Duncan[1] , Anna Schulten[1] , Maria Sindalovskaya[1], Hongchun Yang[1,3], Rebecca Bloomer[1,4], Martin Howard[2,*], Caroline Dean[1,*]

[1] Department of Cell and Developmental Biology, John Innes Centre, Norwich Research Park, Norwich NR4 7UH, UK
[2] Department of Computational and Systems Biology, John Innes Centre, Norwich Research Park, Norwich NR4 7UH, UK
[3] Present address: State Key Laboratory of Hybrid Rice, College of Life Sciences, Wuhan University, Wuhan, 430072, China
[4] Present address: The New Zealand Institute for Plant and Food Research Limited, Christchurch, 8140, New Zealand
†These authors contributed equally to this work

*Corresponding authors

Mathematical model for the dynamics of *FLC* spliced and unspliced transcripts

A simple Ordinary Differential Equation (ODE) model is used to describe the dynamics of spliced and unspliced transcripts. The model assumes that all the unspliced transcripts produced are eventually converted to the spliced form (Fig. S17A). The rate of production of unspliced transcripts is assumed equal to the productive transcription initiation rate. The model equations are as follows:

$$\frac{d[FLC]_{uns}}{dt} = k_{init} - k_{spl}[FLC]_{uns}$$

$$\frac{d[FLC]_{spl}}{dt} = k_{spl}[FLC]_{uns} - k_d[FLC]_{spl}.$$

Here $[FLC]_{uns}$ and $[FLC]_{spl}$ represent the concentration of unspliced and spliced forms respectively. k_{init} represents the productive transcription initiation rate, k_d the degradation rate constant of the spliced form, and k_{spl} the rate constant of unspliced to spliced conversion, i.e., the splicing rate.

At steady state, the spliced and unspliced levels can be computed as follows:

$$[FLC]_{spl} = \frac{k_{init}}{k_d}$$

$$[FLC]_{uns} = \frac{k_{init}}{k_{spl}}.$$

Hence, the spliced to unspliced ratio is given by:

$$R_{spl/uns} = \frac{[FLC]_{spl}}{[FLC]_{uns}} = \frac{k_{spl}}{k_d}.$$

As the degradation rate does not change between cold and non-vernalized (NV) conditions, the change in this ratio between cold conditions and NV can then be used to calculate the fold change in splicing rate between conditions:

$$FC_{spl} = \frac{R^{cold}_{spl/uns}}{R^{NV}_{spl/uns}} = \frac{k^{cold}_{spl}}{k^{NV}_{spl}}.$$

Model including degradation of the unspliced form

To address the possibility of changed degradation of the unspliced *FLC* transcript, we modified the above model to incorporate direct degradation of the unspliced form. (Fig. S17B). The model equations are as follows:

$$\frac{d[FLC]_{uns}}{dt} = k_{init} - k_{spl}[FLC]_{uns} - \gamma[FLC]_{uns}$$

$$\frac{d[FLC]_{spl}}{dt} = k_{spl}[FLC]_{uns} - k_d[FLC]_{spl}$$

where γ represents the degradation rate of the unspliced form. All other parameters represent the same processes as in the simpler model above.

The steady-state levels for the modified model are given by:

$$[FLC]_{uns} = \frac{k_{init}}{k_{spl} + \gamma}$$

$$[FLC]_{spl} = \frac{k_{init}k_{spl}}{k_d(k_{spl} + \gamma)}$$

These expressions can be used to calculate the fold changes in steady-state levels of unspliced and spliced forms between non-vernalized (NV) and cold conditions. We examined three scenarios:

1. Only the degradation rate of the unspliced form is changed in the cold. In this scenario, the fold changes are given by:

$$FC^{unspliced}_{cold} = \frac{k_{spl} + \gamma_{NV}}{k_{spl} + \gamma_{cold}}$$

$$FC_{cold}^{spliced} = \frac{k_{spl} + \gamma_{NV}}{k_{spl} + \gamma_{cold}}$$

Note that the two expressions are identical. Hence, in this scenario the fold changes of spliced and unspliced forms would be expected to be the same, which is inconsistent with the data.

2. Both the transcription initiation rate and the degradation rate of the unspliced form are changed in the cold. In this scenario, the fold changes are given by:

$$FC_{cold}^{unspliced} = \frac{k_{init}^{cold}(k_{spl} + \gamma_{NV})}{k_{init}^{NV}(k_{spl} + \gamma_{cold})}$$

$$FC_{cold}^{spliced} = \frac{k_{init}^{cold}(k_{spl} + \gamma_{NV})}{k_{init}^{NV}(k_{spl} + \gamma_{cold})}$$

Again, the two expressions are identical. Therefore, even in this scenario the fold changes of the spliced and unspliced forms would be expected to be the same, which is inconsistent with the data.

3. The splicing rate, transcription initiation rate and degradation rate of the unspliced form are all changed in the cold. In this scenario, the fold changes are given by:

$$FC_{cold}^{unspliced} = \frac{k_{init}^{cold}(k_{spl}^{NV} + \gamma_{NV})}{k_{init}^{NV}(k_{spl}^{cold} + \gamma_{cold})}$$

$$FC_{cold}^{spliced} = \frac{k_{init}^{cold}k_{spl}^{cold}(k_{spl}^{NV} + \gamma_{NV})}{k_{init}^{NV}k_{spl}^{NV}(k_{spl}^{cold} + \gamma_{cold})}$$

In this scenario the two expressions are different. This difference is realised only because of the change in the splicing rate. This result indicates that a change in the splicing rate is essential to capture the observed differences between the spliced and unspliced forms.

Mathematical model of RNA PolII distribution across the locus

To infer potential changes in PolII speed using the plaNETseq data, we use a simple model, where the *FLC* locus is assumed to be divided into two regions – a gene body region and a termination window at the 3’ end (see schematic in Fig. 4C), with PolII speed being significantly slower in the termination window. The model assumes a constant PolII speed within each window and that all Pol II that initiate productive transcription reach the termination window. The total steady-state PolII within each window is then given by:

$$Pol\ II_{body} = \frac{k_{init}}{v_{body}} l_{body}$$
$$Pol\ II_{term} = \frac{k_{init}}{v_{term}} l_{term}.$$

Here k_{init} represents the productive transcription initiation rate, v_{body} and v_{term} represent the PolII speed in the gene body and termination window respectively, and l_{body} and l_{term} represent the length of the gene body and termination window regions respectively. The termination index (as plotted in Fig. 4C) is given by:

$$Termination\ index = \frac{Pol\ II_{term}}{Pol\ II_{body}} = \frac{v_{body} l_{term}}{v_{term} l_{body}}.$$

The fold change in the termination index relative to the non-vernalized timepoint can be computed as:

$$FC_{Termination\ index} = \frac{Termination\ index_{cold}}{Termination\ index_{NV}} = \frac{v_{body}^{cold} v_{term}^{NV}}{v_{body}^{NV} v_{term}^{cold}}.$$

Hence, if v_{term}, the PolII speed in the termination window, is assumed to be unchanged between conditions, then the fold change in the termination index is equivalent to the fold change in PolII speed within the gene body region:

$$FC_{Termination\ index} = \frac{v_{body}^{cold}}{v_{body}^{NV}}.$$

Mathematical model of *FLC* intron 1 processing

We previously developed a mathematical model to capture variation in steady state RNA levels across an intron, incorporating the effects of productive transcription initiation, PolII speed, intron processing, and intron lariat degradation, and applied it to *FLC* intron 1 [1]. The model also incorporated differences between intronic RNA levels measured in total RNA and chromatin-bound fractions – by assuming that the total RNA fraction included contributions from the intron lariat, in addition to the entire chromatin bound fraction. By combining this model with qPCR measurements of RNA levels tiling across *FLC* intron 1 and examining both chromatin-bound and total RNA fractions, we were able to reveal differences in the initiation rate and PolII speed underlying different transcriptional states of the *FLC* locus. Our analysis revealed that the transcriptional differences between an active state of *FLC* in the mutant *fca-9* and a Polycomb silenced state of *FLC* in the wild-type Col-0 involved a coordinated decrease in both initiation rate and PolII speed.

The model assumes that steady state RNA levels along an intron consist of three contributing fractions: (i) nascent RNA from PolII currently transcribing through the

intron, (ii) nascent RNA from PolII that have transcribed past the 3' splice site at the end of the intron, but where the intron lariat has not started being degraded, (iii) intron lariat RNA currently being degraded, see Fig. 4A. The total nascent RNA contribution ((i) and (ii) above) can be computed as:

$$\text{Intron 1 Nascent RNA}(x) = \int_{x}^{\text{IA}} \text{PolII}(y)\, dy + \text{RNA}_\text{s}$$

where PolII(y) represents the density of PolII at location y, the variable *x* represents position relative to the *FLC* transcription start site (*x*=0 at the TSS), and IA represents the position of the intron acceptor site relative to the TSS. RNA_s represents the contribution from PolII that have transcribed past the 3' end of the intron ((ii) above).

Assuming a constant PolII speed along the intron and no termination within the intron, PolII(y) will be equal to F/v, where F represents the productive transcription initiation rate (more specifically, the frequency of PolII transcribing into the intron), and v represents the PolII speed across the intron. The contribution RNA_s is equal to F/k_s, since these transcripts are being produced at a rate F and their contribution to intronic RNA is being removed by the process of splicing followed by lariat degradation. Here the parameter k_s represents the intron processing rate. The spliced intronic RNA is not immediately available for lariat degradation, since it has to first undergo further steps including debranching. Taking into account the time taken for these intermediate steps between intron cleavage and the start of lariat degradation, we note that the intron processing rate k_s in the above model does not just represent the rate of cleavage of the intron at its 3' splice site. Rather k_s in the model is a composite parameter representing the rate of the whole process through which intronic RNA that is initially part of the nascent transcript goes on to become intron lariat RNA subject to degradation. The nascent RNA contribution at a location x within the intron can thus be computed as:

$$\text{Intron 1 Nascent RNA}(x) = F\left(\frac{IA - x}{v} + \frac{1}{k_s}\right)$$

Similarly, the lariat contribution to intronic RNA levels can be computed as follows:

$$\text{Intron 1 Lariat RNA}(x) = F\left(\frac{x - ID}{k_d}\right)$$

assuming a constant rate of lariat degradation in the 5' to 3' direction, with a degradation rate of k_d (bp/s). ID represents the position of the intron donor site relative to the TSS.

The resulting equation for the Wu et al. 2016 [1], model was as follows:

$$\text{Intron 1 RNA}(x)_{\text{Total}} = F\left(\frac{IA-x}{v}+\frac{1}{k_s}+\left(\frac{x-ID}{k_d}\right)\right)$$

for the total RNA fraction.

As discussed above, the parameter k_s is not equivalent to the splicing rate whose fold change was estimated in the main paper using the spliced to unspliced ratio. This measurement was made via an intron-exon junction assay, and so may be directly interpreted as the rate of cleavage at the 3' splice site. However, the two rate measurements – splicing rate and intron processing rate both give consistent increases in the cold versus NV, albeit for different introns, thereby giving confidence that splicing rates are generally increased at *FLC* in the cold.

It is also possible to include 3'-5' lariat degradation in the above model. However, in the current study, introducing 3'-5' lariat degradation at a similar rate to the 5'-3' degradation was found to make the model fit badly. Even in the best fit model, it resulted in a pronounced reduction in the predicted profile from the middle of the intron towards the 3' end. Since the data does not exhibit such a trend, we assume that any 3'-5' lariat degradation must be happening at a much lower rate than 5'-3' degradation. We previously estimated lariat degradation rate using an smFISH based quantitative approach in Ietswaart et al., 2017 [2], where we allowed for degradation in both directions, and the estimated rates were comparable for 5'-3' and 3'-5' degradation. However, in Wu et al. 2016 [1], we found that 3'-5' degradation could be comparable but had to be slower than 5'-3' degradation to allow good model fits to the intron tiling fold change profiles between high transcriptional and silenced states of *FLC*. The reason the model fits in Wu et al. 2016 [1] can accommodate comparable 3'-5' degradation is that there are large fold changes in initiation (~25 fold) and PolII speed (~10 fold) between active and silenced states. In the current study, PolII speed changes (up to ~1.5 fold) and initiation rate changes (up to ~2 fold) between warm and 2W, 4W cold. This makes the model predictions more sensitive to the presence of significant 3'-5' lariat degradation. We therefore neglect it in the model.

The Wu et al. 2016 model assumed that the intron lariat RNA was completely released from the chromatin, i.e., lariat RNA did not contribute to the chromatin-bound RNA fraction in this model. The equation for the intron 1 chromatin-bound RNA was therefore as follows:

$$\text{Intron 1 RNA}(x)_{Chr} = F\left(\frac{IA-x}{v}+\frac{1}{k_s}\right).$$

For this equation to be correct, the intron needs to be cleaved at the 3' splice site before the RNA is released from the locus. According to previous estimates of PolII transcription speed across *FLC* [1, 2], and the subsequent rate of transcript release from the locus, the time available between transcription of the intronic RNA and RNA release from the locus is on the order of 30 min. Indeed, the reported timescales for

cleavage of introns is of the order of tens of seconds across different organisms [3], and earlier estimates for *FLC* are around 5 min [2].

We first used the same model and the same methodology as Wu et al. 2016 [1], using intron 1 to examine cold-induced transcriptional repression at *FLC*. However, as explained in the Results section, this model could not capture the experimentally observed trends for RNA levels across intron 1 in both chromatin-bound and total RNA fractions. Specifically, the experimentally measured fold-change profiles (relative to NV samples) in both fractions exhibit an increasing trend from the 5' to the 3' end of the intron. The Wu et al., 2016 model could not capture these trends through a coordinated reduction only in initiation rate and PolII speed (Fig. S6). However, through an increased intron processing rate in the cold combined with reduced initiation rate, the model could capture the 5' to 3' trend for the total RNA fraction. The model was still unable to capture the trend for the chromatin-bound RNA fractions. Importantly, this trend in the experimental data is consistent across cold time-points, and for both total RNA and chromatin-bound RNA fractions.

We therefore developed a modified model by introducing an additional component into the Wu et al. 2016 model – the possibility of partial lariat retention and degradation in the chromatin-bound RNA fraction. Thus, in the modified model, the equation for the total RNA fraction remains the same, while the equation for the chromatin-bound RNA fraction is as follows:

$$\text{Intron 1 RNA}(x)_{\text{Chr}} = F\left(\frac{IA - x}{v} + \frac{1}{k_s} + \text{f}_{\text{lariat}}\left(\frac{x - ID}{k_d}\right)\right),$$

where f_{lariat} is a parameter constrained to be between 0 and 1, representing the partial contribution from the intron lariat component to the chromatin-bound RNA fraction.

Model Fitting and Parameter Inference

Similar to Wu et al. 2016, where we performed quantitative fitting of the model to fold changes in RNA levels between mutants and a wild-type, here we perform quantitative fits to fold changes between time points, specifically between each cold time point and the non-vernalized (NV) time point.

For fitting the intron 1-fold change profiles to both chromatin-bound and total RNA fraction datasets, we fix the basal absolute values of all model parameters corresponding to the non-vernalized (NV) time point and use parameter inference to estimate the fold changes of specific parameters at the cold-treated time points. Thus, the model used for parameter inference is as follows:

$$\text{Fold}(x)_{\text{Total}} = \frac{\text{fc}_{\text{init}}(\text{t})F_{\text{NV}}\left(\frac{IA - x}{v_{\text{cold}}} + \frac{1}{\text{fc}_{\text{proc}}(\text{t})k_s(\text{NV})} + \left(\frac{x - ID}{k_d}\right)\right)}{F_{\text{NV}}\left(\frac{IA - x}{v_{\text{NV}}} + \frac{1}{k_s(\text{NV})} + \left(\frac{x - ID}{k_d}\right)\right)}$$

for the total RNA fraction, and

$$\text{Fold}(x)_{\text{Chr}} = \frac{\text{fc}_{\text{init}}(\text{t})F_{\text{NV}}\left(\frac{IA-x}{v_{\text{cold}}} + \frac{1}{\text{fc}_{\text{proc}}(\text{t})k_s(\text{NV})} + f_{\text{lariat}}\left(\frac{x-ID}{k_d}\right)\right)}{F_{\text{NV}}\left(\frac{IA-x}{v_{\text{NV}}} + \frac{1}{k_s(\text{NV})} + f_{\text{lariat}}\left(\frac{x-ID}{k_d}\right)\right)}$$

for the chromatin-bound RNA fraction. Here, the parameters fc_{init} and fc_{proc} represent the fold changes in the productive initiation rate and intron processing rate at a given time point relative to the NV values of these rates, see Fig. 4B,C.

Throughout this analysis productive transcription initiation appears only as a fold change between experimental conditions – we therefore do not assume an absolute value for this parameter. PolII speed at *FLC* corresponding to non-vernalized Col*FRI* is assumed to be *v*= 40 bp/s, the estimated mean value for a high transcriptional state of *FLC* from Wu et al. 2016 [1]. Using the median PolII speed estimated in Ietswaart et al., 2017 (11 bp/s), the model could not qualitatively fit the trends in our data. The basal intron processing rate is fixed at 0.002 s^{-1}, consistent with the estimate for this parameter from Ietswaart et al., 2017 [2]. Note that, as discussed above, this parameter represents a composite of multiple steps in the model, and therefore should not be treated as equivalent to the rate of intron cleavage at the 3' splice site. The basal 5'-3' degradation rate is assumed to be 3 bp/s, the estimated median value of this parameter from Ietswaart et al., 2017. The inferred parameters for the modified model were the fold changes in initiation and intron processing rates (fc_{init} and fc_{proc}), with a fold change reduction in PolII speed that changes over the cold timepoints as described in the text. This is consistent with a cold-induced slowdown suggested by our plaNETseq data (termination index in Fig. 3C), previous quantitative analysis of co-transcriptionally added H3K36me3 [4], and the estimated difference in PolII speed between an active transcriptional state and a Polycomb silenced (H3K27me3 spread) state [1]. The f_{lariat} parameter, representing the lariat component contribution to the chromatin-bound fraction, was inferred using data for the 2 week time point, and held fixed at the estimated value (0.17) for all other time points. Allowing the lariat degradation rate to change in the cold (along with the above inferred fold changes), and inferring this change as an additional parameter, predicted an increase in the lariat degradation rate in the cold, however without significantly improving the model fits (fitted models compared using the Bayesian evidence – see below). We therefore keep the lariat degradation rate fixed at its basal value for all time points. Parameter values used for the *FLC* intron 1 processing model are also shown in Table S1.

We used a Bayesian approach for model parameter inference [5], using a nested sampling algorithm [6] implemented in the Python package Nestle [7]. This procedure is described in the Methods.

RNA decay analysis with non-zero asymptote

To examine the possibility that the ActD treatment may have only partially shut-down transcription, we fit an alternative model to the smFISH decay data presented in the manuscript (Fig. 2B). The alternative model comprises an exponential decay with a

non-zero asymptote, which can capture any residual transcription. We inferred the three parameters – starting level, decay rate constant, and residual level – using the nested sampling approach described in the Methods. For both non-vernalized and 2-week cold treated conditions, the posterior probability for the residual level peaked around zero, indicating that the ActD treatment has been effective in shutting-down transcription in both conditions (Fig. S15, S16).

References

1. Wu, Z., et al., *Quantitative regulation of FLC via coordinated transcriptional initiation and elongation.* Proc Natl Acad Sci U S A, 2016. **113**(1): p. 218-23.
2. Ietswaart, R., et al., *Cell-Size-Dependent Transcription of FLC and Its Antisense Long Non-coding RNA COOLAIR Explain Cell-to-Cell Expression Variation.* Cell Syst, 2017. **4**(6): p. 622-635 e9.
3. Alpert, T., L. Herzel, and K.M. Neugebauer, *Perfect timing: splicing and transcription rates in living cells.* Wiley Interdiscip Rev RNA, 2017. **8**(2).
4. Nielsen, M., et al., *COOLAIR and PRC2 function in parallel to silence FLC during vernalization.* Proc Natl Acad Sci U S A, 2024. **121**(4): p. e2311474121.
5. Pullen, N. and R.J. Morris, *Bayesian model comparison and parameter inference in systems biology using nested sampling.* PLoS One, 2014. **9**(2): p. e88419.
6. Skilling, J., *Nested sampling.* Bayesian inference and maximum entropy methods in science and engineering, 2004. **735**: p. 395-405.
7. Barbary, K., *nestle: Pure Python, MIT-licensed implementation of nested sampling algorithms for evaluating Bayesian evidence.* 2014.

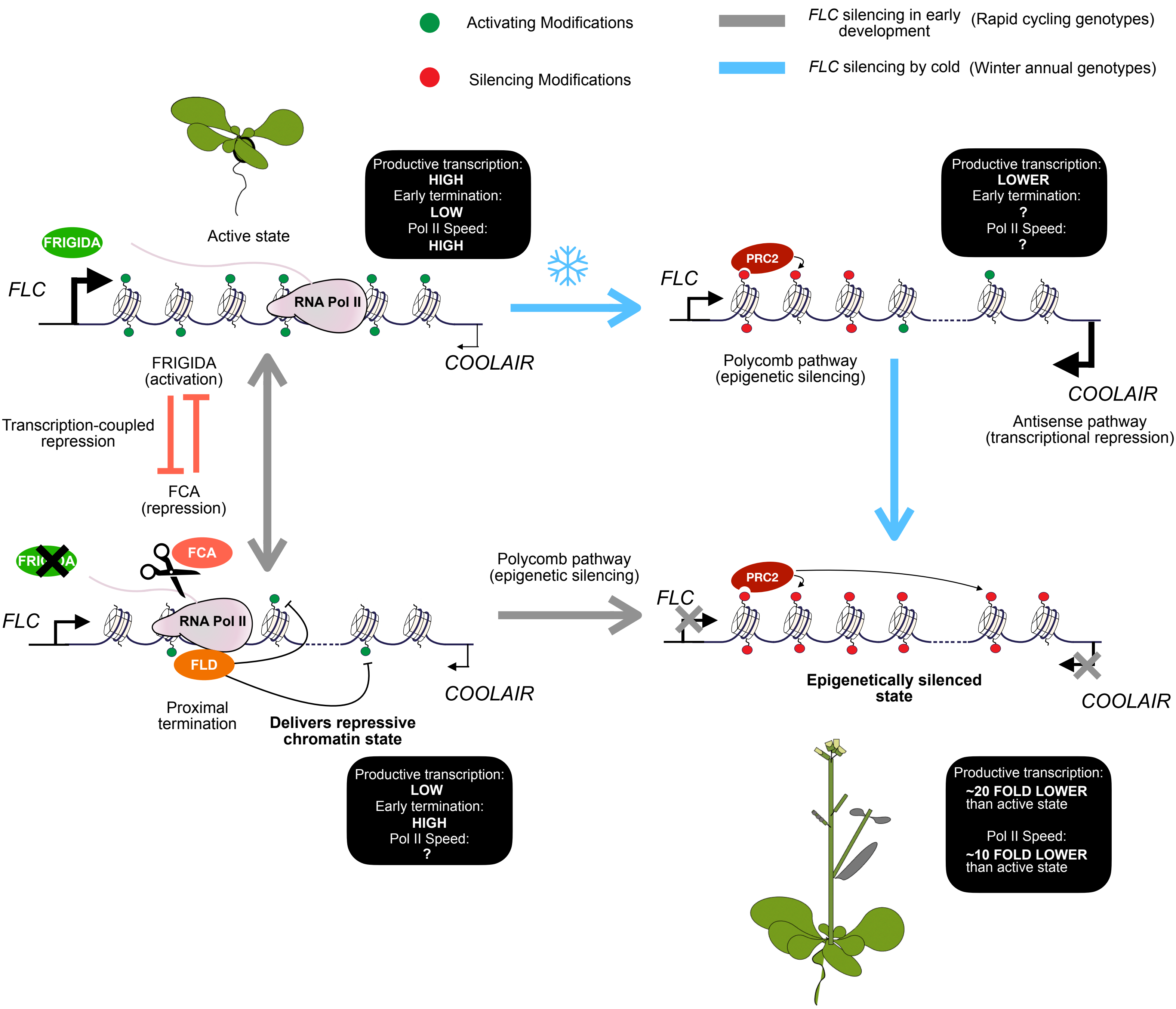

Figure S1: Schematic summary of transitions in *FLC* transcriptional state and the molecular mechanisms regulating these transitions.

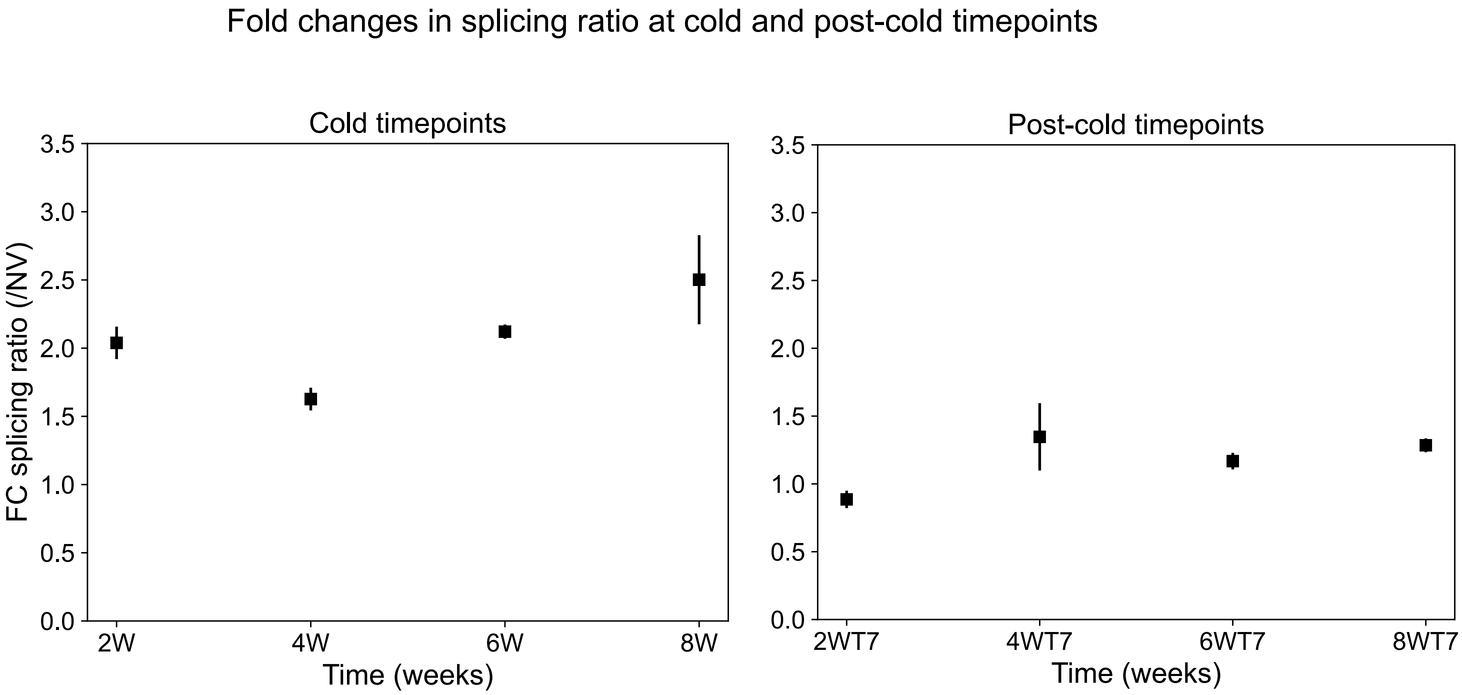

Figure S2: Fold changes in splicing ratio calculated for all cold and post-cold timepoints. The fold changes were calculated using the data shown in Figure 1A. See model description for the ODE model showing how this fold change can represent the fold change in splicing rate at these timepoints, relative to the non-vernalised condition (NV). Error bars represent SEM (n=3 biological replicates).

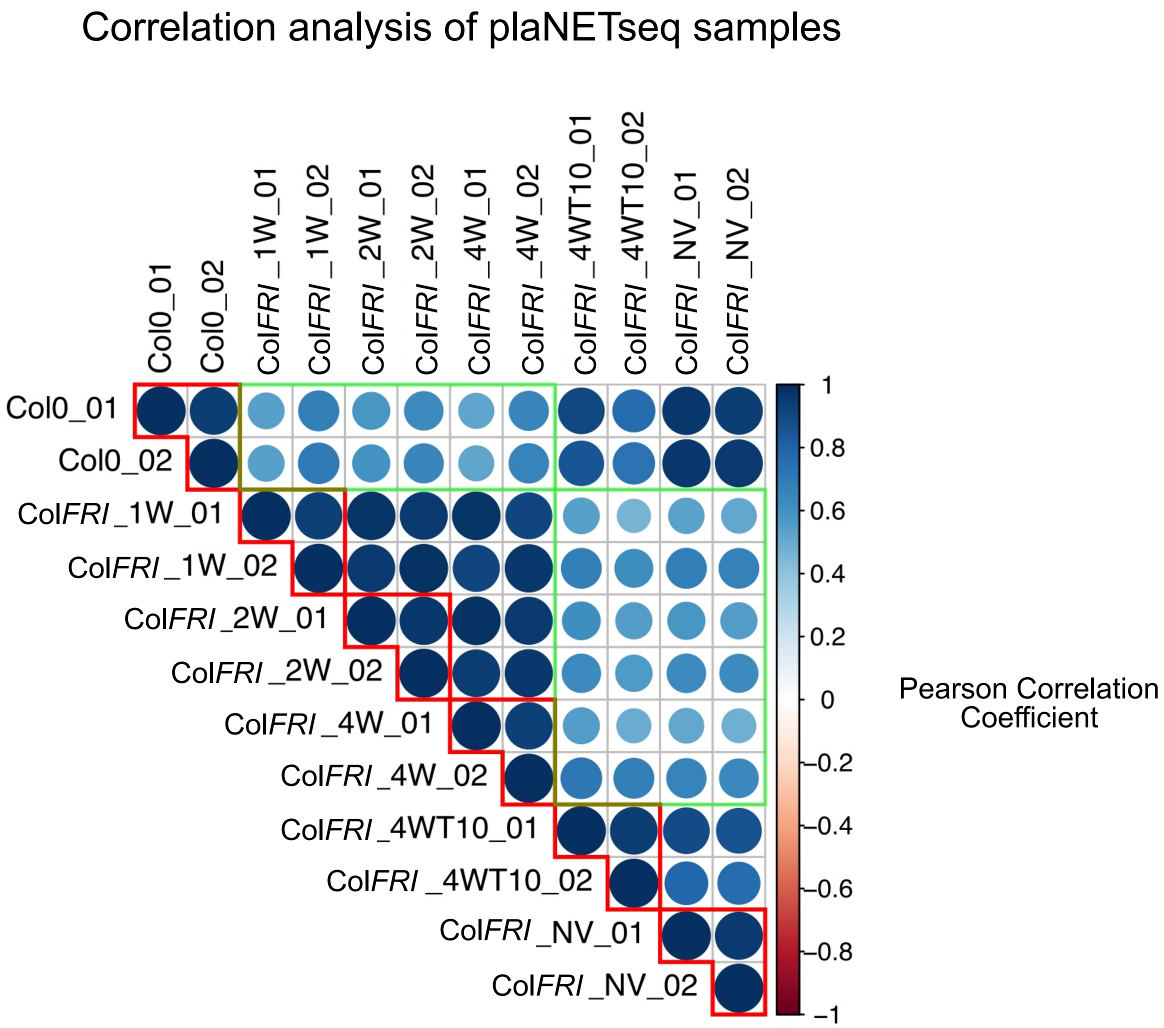

Figure S3: Correlation analysis of plaNETseq reads after in solution target capture. Colour intensity and the size of the circle are proportional to the Pearson correlation coefficients between each pair of samples. Red boxes indicate replicate comparisons and green boxes highlight NV and Cold library comparisons. Library correlation remains high for replicates and is reduced when comparing treatment groups.

Normalized plaNETseq counts of control genes

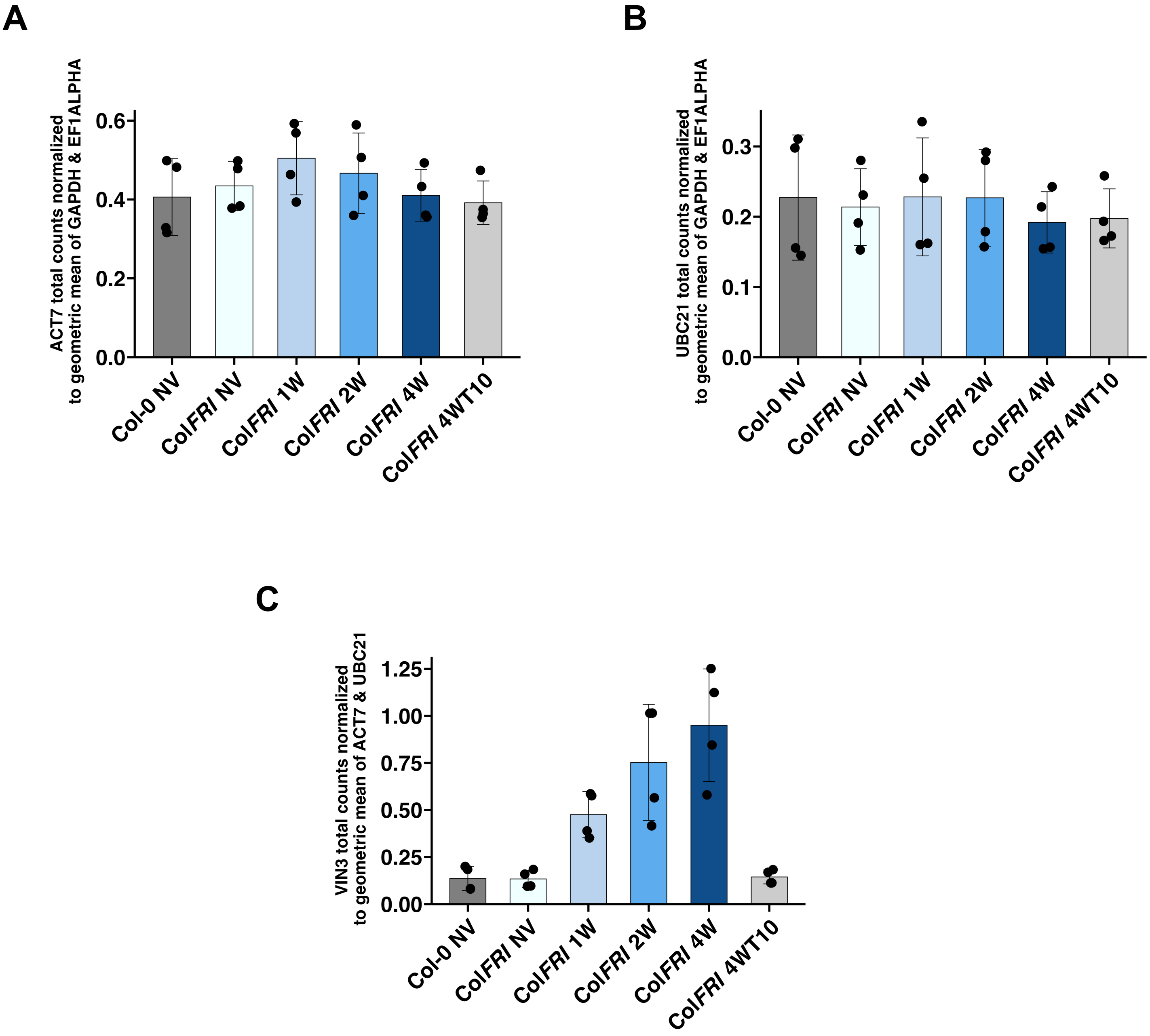

Figure S4: plaNETseq levels of control genes. Total plaNET-seq counts of bait-enriched control genes. Mean counts of 4 biological replicates of *ACT7* (A) and *UBC21* (B), normalized to the geometric mean of *GAPDH* and *EF1ALPHA*, and *VIN3* (C), normalized to the geometric mean of *ACT7* and *UBC21*.

Normalized plaNETseq profile at *FLC*

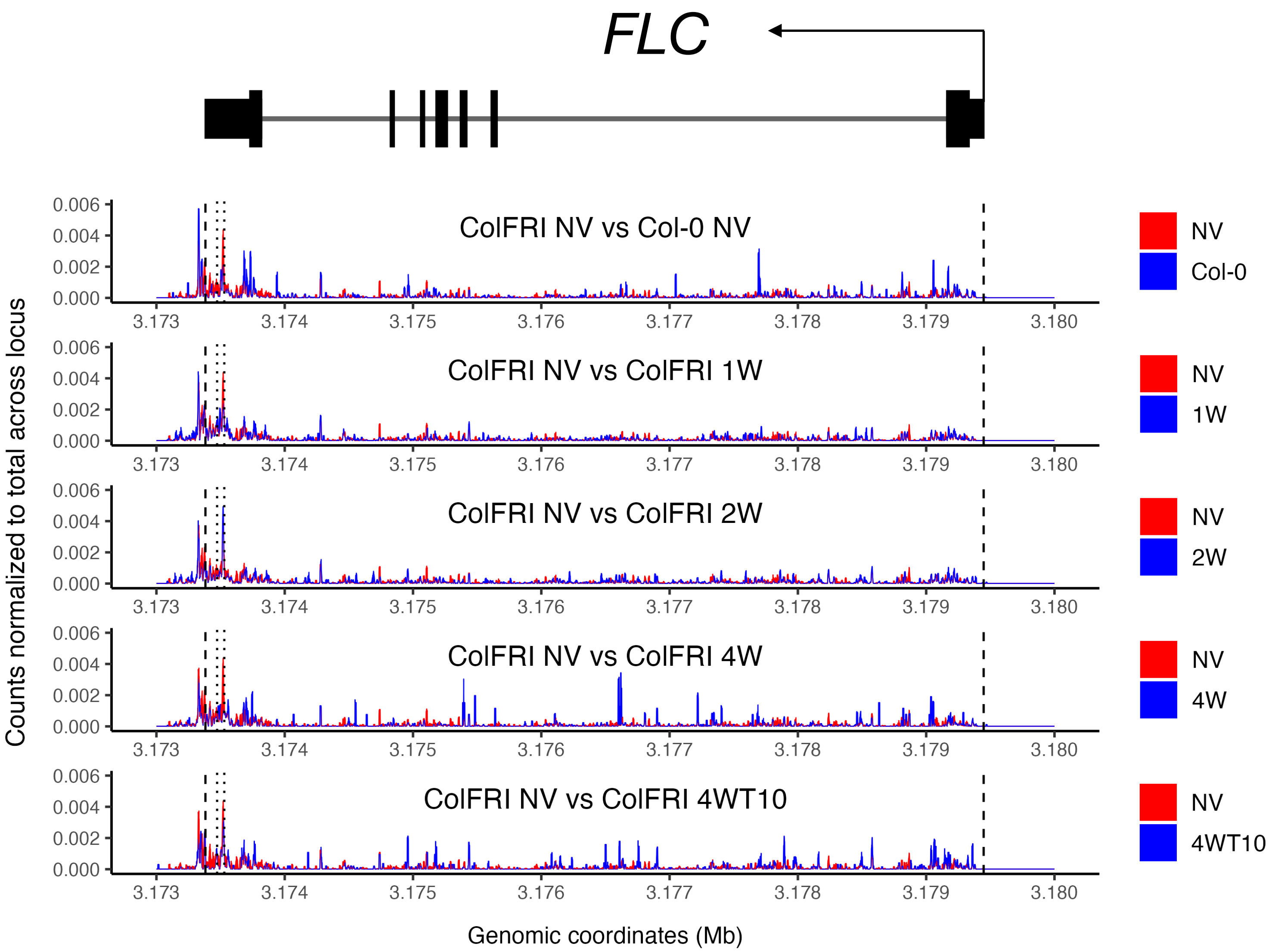

Figure S5. plaNETseq analysis of the *FLC* locus over a cold time course. Normalized PolII distributions across *FLC* over the vernalization time series. Mean counts of 4 biological replicates was normalized to the total PolII levels across the locus. Each panel shows a comparison between a cold timepoint and NV conditions in Col*FRI* or between Col-0 NV and Col*FRI* NV. Distributions are plotted as curves and the space between the curves is shaded blue where the cold timepoint or Col-0 NV shows higher normalized PolII level and red where the Col*FRI* NV timepoint shows higher normalized PolII level. *FLC* TSS and the end of 3' UTR (Araport 11 annotation) are represented by black dashed lines and major poly-A sites are shown by black dotted lines. (B) Total plaNETseq counts over *FLC* over the cold timecourse.

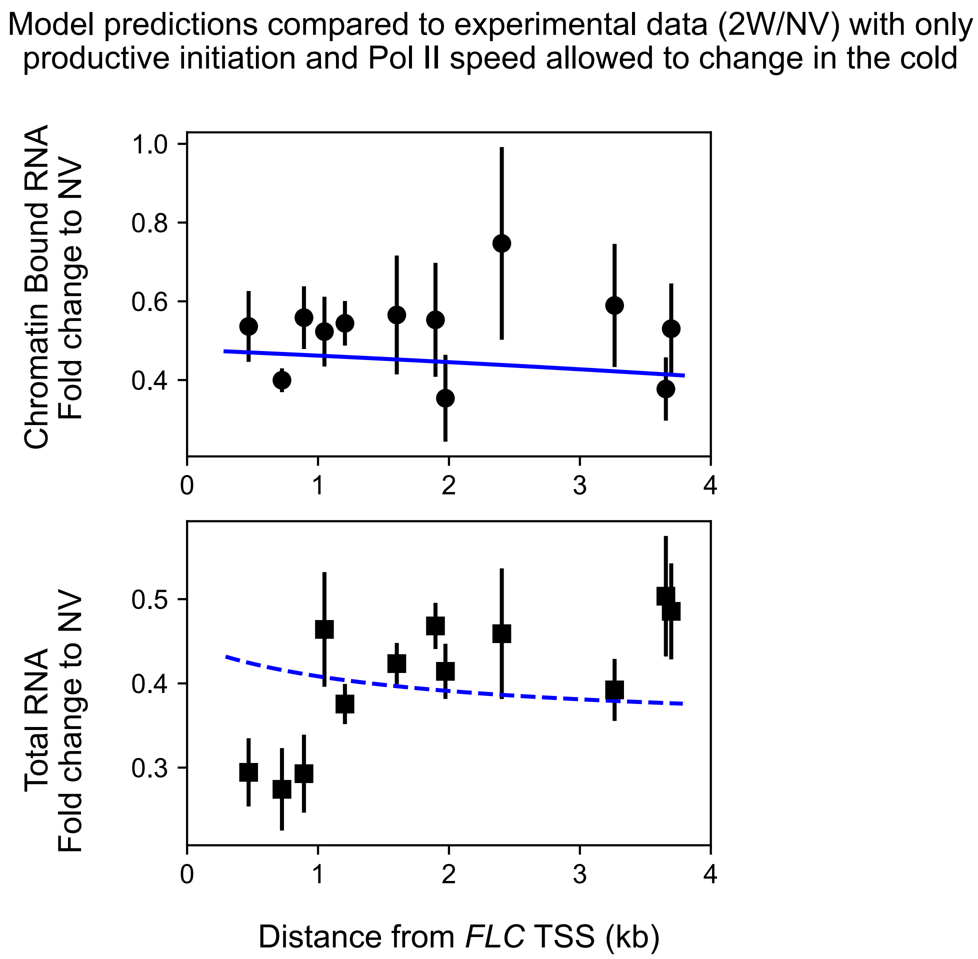

$$FC_{init\ total} = 0.38 \quad FC_{init\ chr} = 0.41 \quad FC_{speed} = 0.5$$

Figure S6: Model fails to fit measured profiles with only initiation and Pol II speed changes. Model predictions indicated by blue lines – chromatin bound RNA by solid line and total RNA by dashed line. Experimental datapoints indicated by black dots (chromatin bound RNA) and black squares (total RNA). With only coordinated reduction in initiation and PolII speed, the model fails to even qualitatively capture the trend in the total RNA profile over intron 1 at 2W relative to NV. Best fit shown, with parameter values for fold changes in productive initiation and PolII speed shown. Error bars represent SEM (n=3 biological replicates).

Parameter inference for Wu et al., 2016 *FLC* Intron 1 model using
only Total RNA Intron 1 tiling data (2W relative to NV)

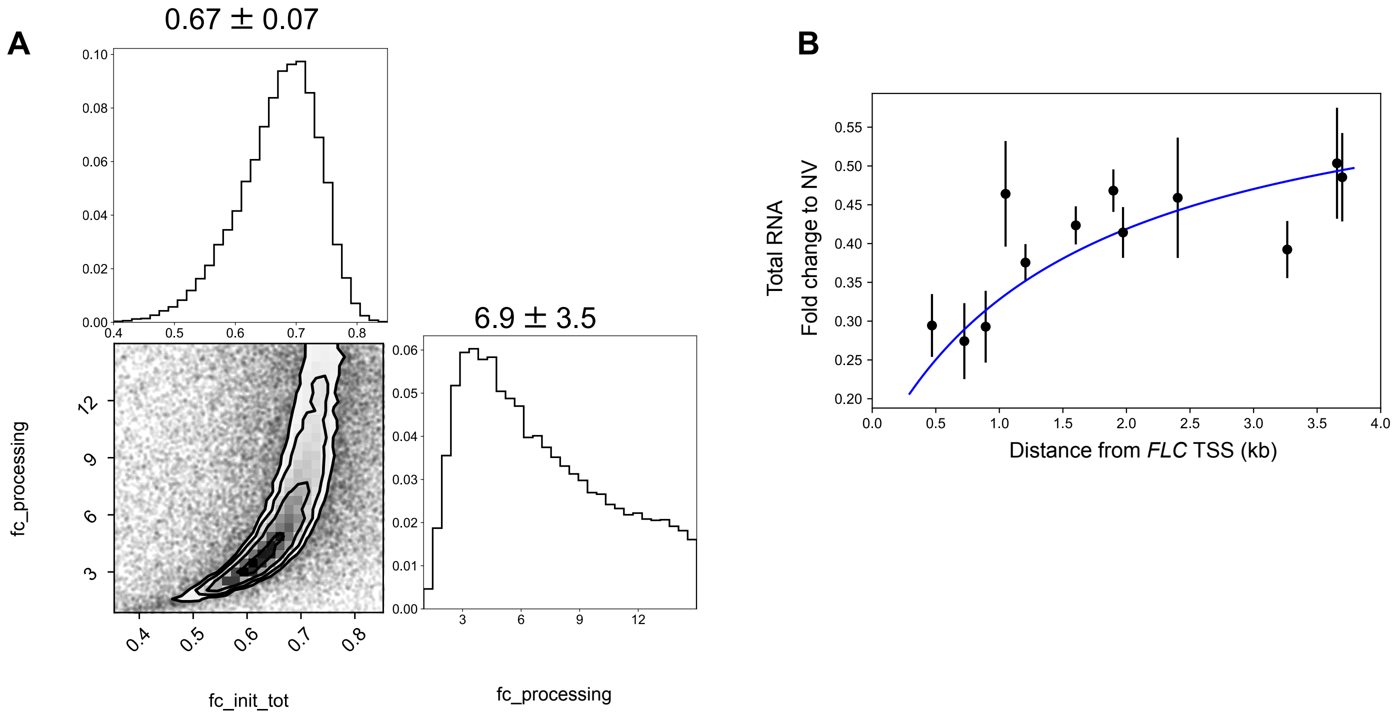

Figure S7. Estimated posterior distribution of the inferred parameters, obtained using a nested sampling approach, from 2W timepoint Total RNA data. (A) Two model parameters were inferred using data for the 2W time point. Total RNA data was used to simultaneously constrain the model, and PolII speed was assumed to change by a factor of 0.8 at 2W cold. Individual contour plots show two dimensional projections of the final set of parameter samples generated by the nested sampling algorithm. The histograms show the posterior probability of the corresponding parameter values. Mean ± standard deviation of the posterior samples is shown for each parameter. The parameter names are as follows: fc_init_tot denote fold changes in initiation (2W/NV) estimated from the total RNA profile; fc_processing denotes the fold change in intron processing rate (2W/NV); Uniform priors were assumed for both parameters, within the following bounds: 0<fc_init_tot<2; 0<fc_processing<15. (B) Model fits using the mean values of the posterior sample distributions for both parameters.

Parameter inference for *FLC* Intron 1 model using Intron 1 tiling data
(2W relative to NV)

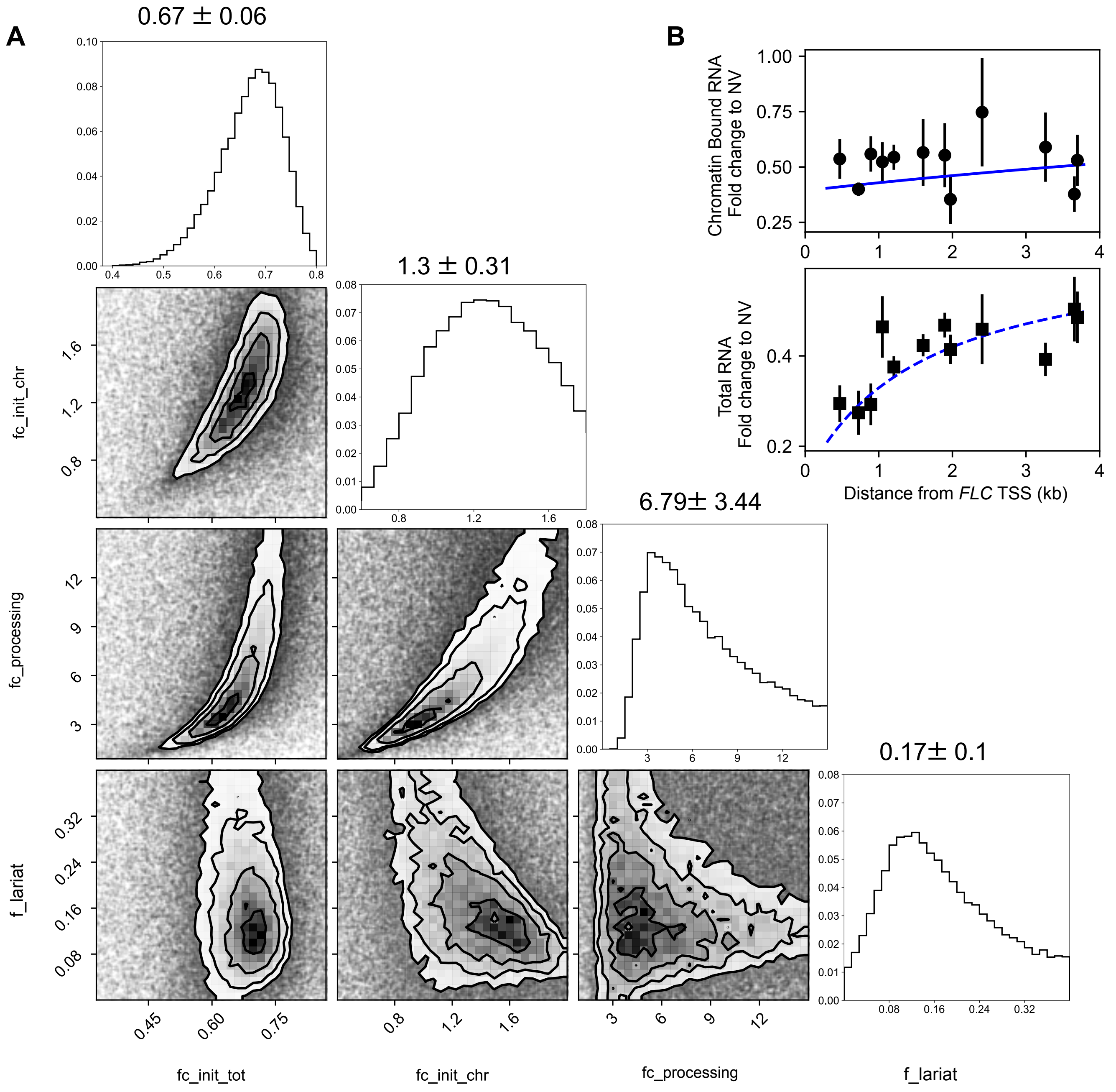

Figure S8. Distribution of sampled parameter values, sampled from the posterior distribution, using a nested sampling approach, using 2W time point data. (A) Four model parameters were inferred using data for the 2W time point. Chromatin-bound RNA data and total RNA data were used to simultaneously constrain the model, and PolII speed was assumed to change by a factor of 0.8 at 2W cold. Individual contour plots show two dimensional projections of the final set of parameter samples generated by the nested sampling algorithm. The histograms show the posterior probability of the corresponding parameter values. Mean ± standard deviation of the posterior samples is shown for each parameter. The parameter names are as follows: fc_init_tot and fc_init_chr denote fold changes in initiation (2W/NV) estimated from the total RNA and chromatin-bound RNA profiles, respectively; fc_processing denotes the fold change in intron processing rate (2W/NV); f_lariat denotes the fractional component of lariat contributing to the chromatin-bound RNA fraction. Uniform priors were assumed for all four parameters, within the following bounds: 0<fc_init_tot<2; 0<fc_init_chr<2; 0<fc_processing<15; 0<f_lariat<0.4. (B) Model fits using the mean values of the posterior sample distributions for all four parameters (reproduced from Fig. 4).

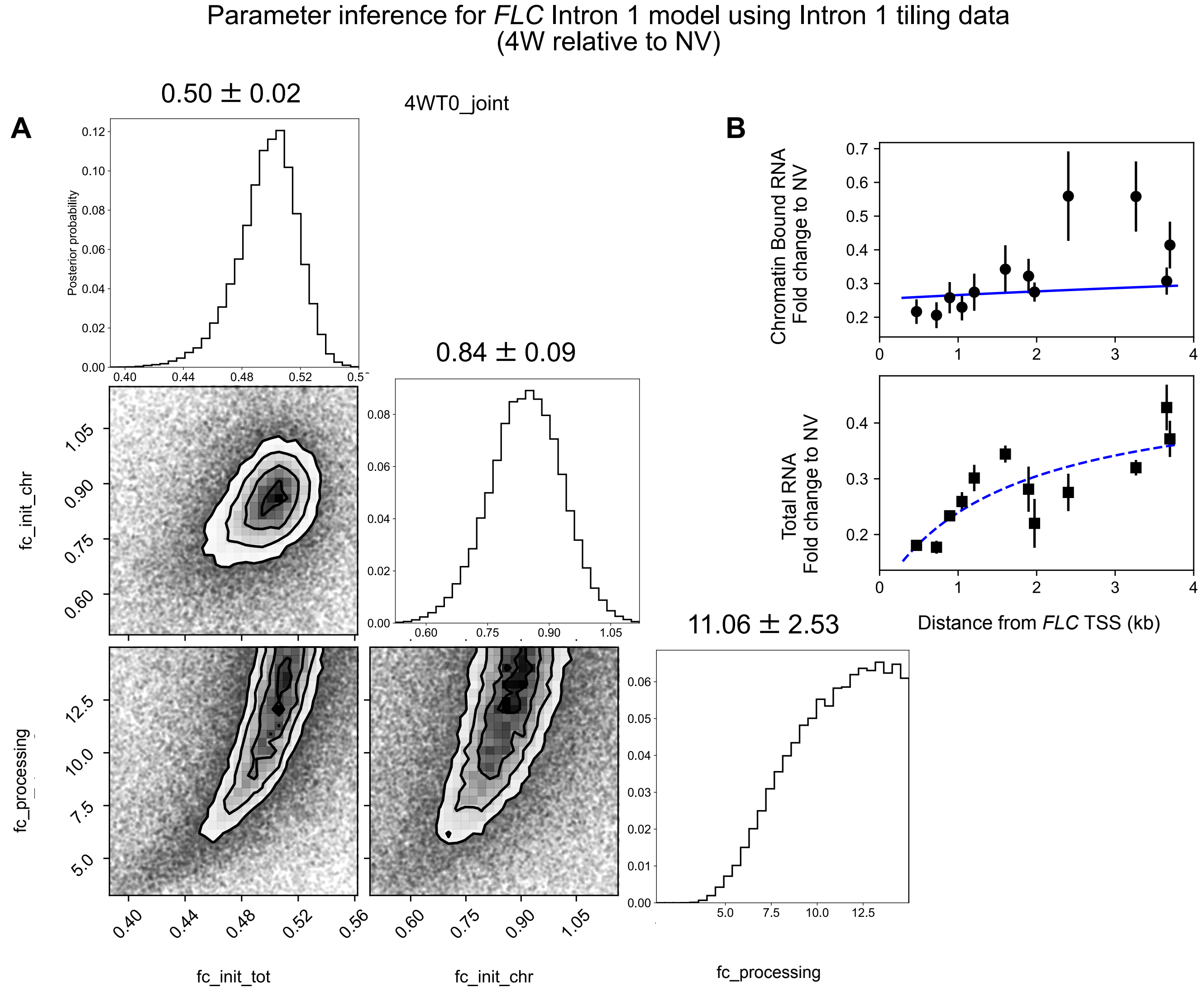

Figure S9. Distribution of sampled parameter values, sampled from the posterior distribution, using a nested sampling approach, using 4W time point data. (A) Three model parameters were inferred using data for the 4W time point. Chromatin-bound RNA data and total RNA data were used to simultaneously constrain the model and PolII speed was assumed to change by a factor of 0.65 at 4W cold. Individual contour plots show two dimensional projections of the final set of parameter samples generated by the nested sampling algorithm. The histograms show the posterior probability of the corresponding parameter values. Mean ± standard deviation of the posterior samples is shown for each parameter.The parameter names are as follows: fc_init_tot and fc_init_chr denote fold changes in initiation (4W/NV) estimated from the total RNA and chromatin-bound RNA profiles, respectively; fc_processing denotes the fold change in intron processing rate (4W/NV); f_lariat was fixed at 0.17 (the mean value of the posterior distribution estimated from the 2W data). Uniform priors were assumed for all three parameters, within the following bounds: 0<fc_init_tot<2; 0<fc_init_chr<2; 0<fc_processing<15. (B) Model fits using the mean values of the posterior sample distributions for all three parameters (reproduced from Fig. 4).

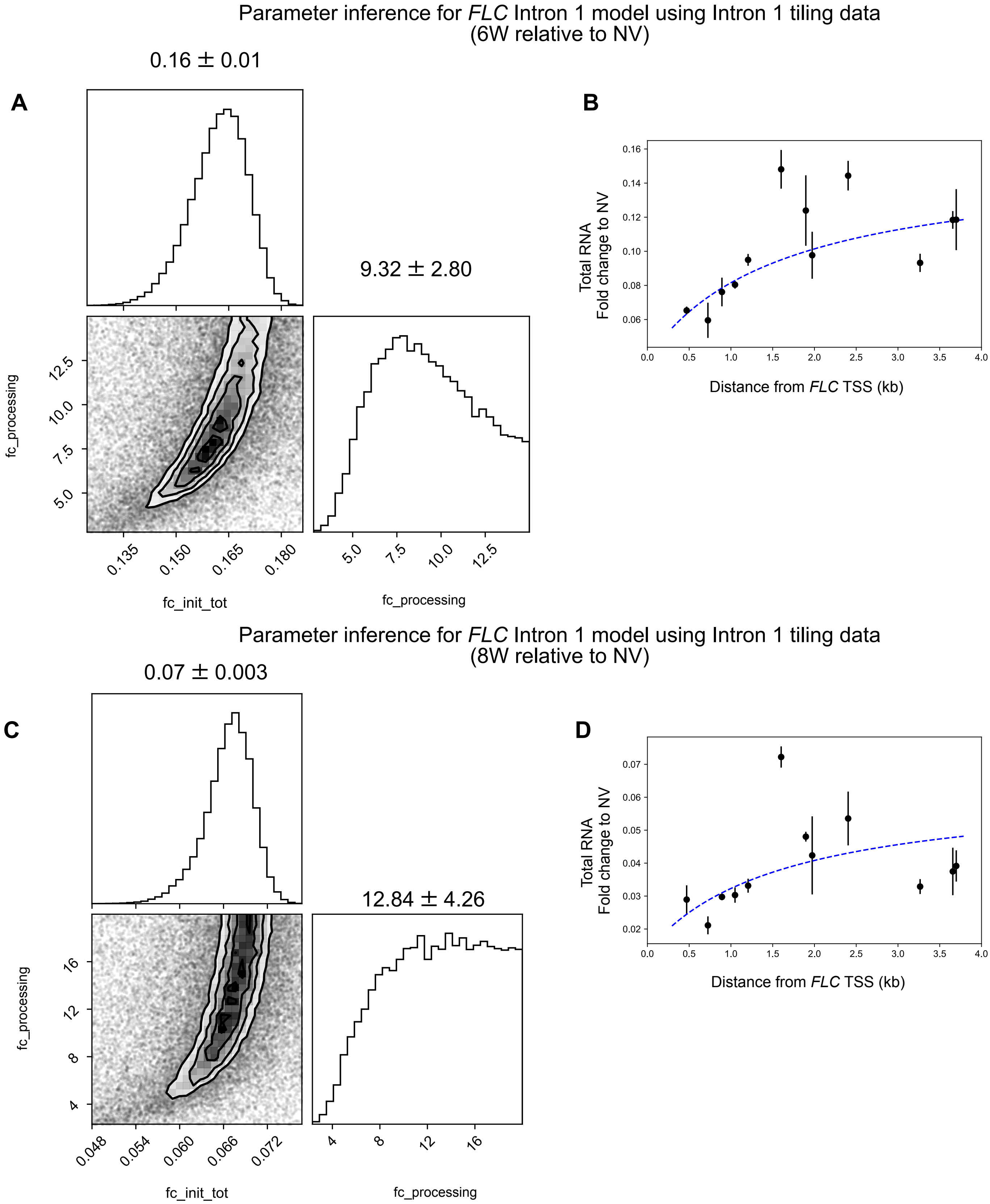

Figure S10. Distribution of sampled parameter values, sampled from the posterior distribution, using a nested sampling approach, using 6W and 8W time points data. (A,C) Two model parameters were inferred using data for the 6W and 8W time points. Total RNA data was used to constrain the model, and PolII speed was assumed to change by a factor of 0.6 relative to NV. Individual contour plots show two dimensional projections of the final set of parameter samples generated by the nested sampling algorithm. The histograms show the posterior probability of the corresponding parameter values. Mean ± standard deviation of the posterior samples is shown for each parameter.The parameter names are as follows: fc_init_tot denotes fold changes in initiation (relative to NV), estimated from the total RNA profile; fc_processing denotes the fold change in intron processing rate (relative to NV); f_lariat was fixed at 0.17 (the mean value of the posterior distribution estimated from the 2W data). Uniform priors were assumed for both parameters, within the following bounds: 0<fc_init_tot<2; 0<fc_processing<15. (B, D) Model fits (dashed blue curves) using the mean values of the posterior sample distributions for the two parameters.

Parameter inference for *FLC* Intron 1 model using Intron 1 tiling data
(Post-cold relative to NV)

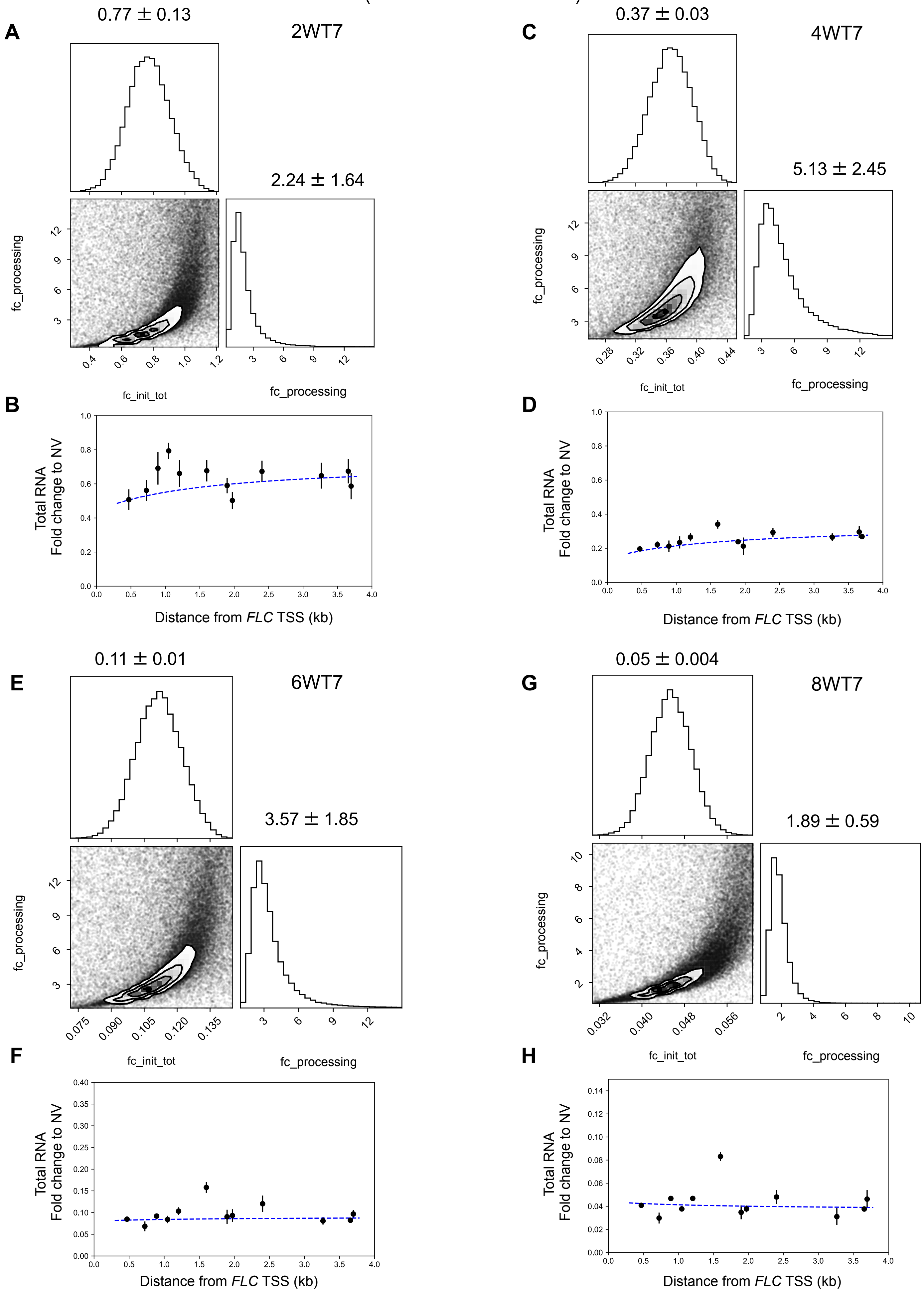

Figure S11. Distribution of sampled parameter values, sampled from the posterior distribution, using a nested sampling approach, using data for all post-cold time points. (A, C, E, G) Two model parameters were inferred using data for all post-cold time points: 2WT7, 4WT7, 6WT7, 8WT7. Total RNA data was used for parameter inference, and PolII speed is assumed to change by the following factor relative to NV: 0.6 at 2WT7, 0.5 at 4WT7, 0.3 at 6WT7 and 8WT7. Individual contour plots show two dimensional projections of the final set of parameter samples generated by the nested sampling algorithm. The histograms show the posterior probability of the corresponding parameter values. Mean ± standard deviation of the posterior samples is shown for each parameter.The parameter names are as follows: fc_init_tot denotes fold changes in initiation (relative to NV), estimated from the total RNA profile; fc_processing denotes the fold change in intron processing rate (relative to NV); f_lariat was fixed at 0.17 (the mean value of the posterior distribution estimated from the 2W data). Uniform priors were assumed for both parameters, within the following bounds: 0<fc_init_tot<2; 0<fc_processing<15. (B, D, F, H) Model fits (dashed blue curves) using the mean values of the posterior sample distributions for the two parameters.

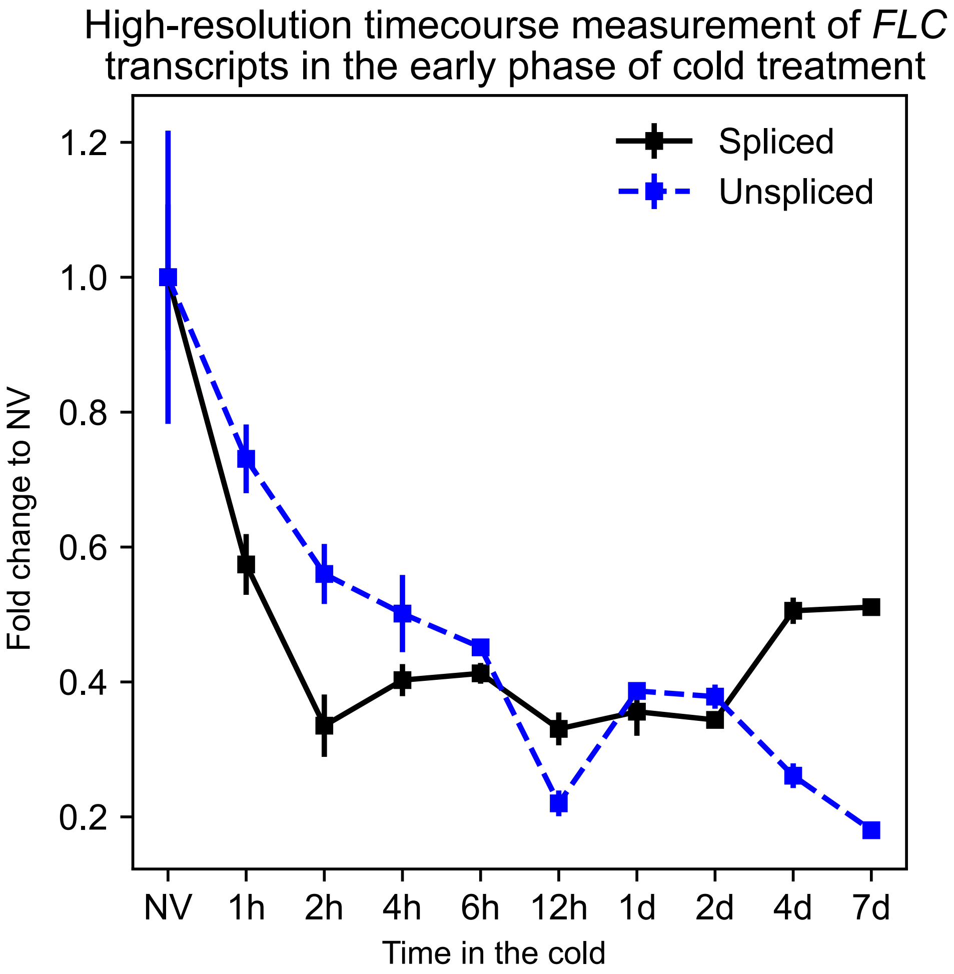

Figure S12. Fold changes in cold induced reduction of spliced and unspliced *FLC* transcripts show opposite trends over short and long timescales. Relative expression of spliced and unspliced *FLC* (as defined in the Results section – also see list of primers) over a vernalization time course (constant 5°C) in Col*FRI*. Values are normalized to the housekeeping gene UBC and to the NV levels. Error bars represent SEM (n=4 for NV timepoint, n=3 for all other timepoints). Over the first 6h of cold treatment, spliced *FLC* level reduces faster than unspliced *FLC.* This trend is reversed over the 2 to 7 day phase, where unspliced *FLC* level reduces and spliced *FLC* level shows no further reduction. This is consistent with the long term trend observed in Fig. 1(A), where the relative reduction in unspliced *FLC* is significantly greater than for spliced *FLC* over 2, 4, 6 and 8 week timepoints.

FLC mRNA decay rate quantification by smFISH

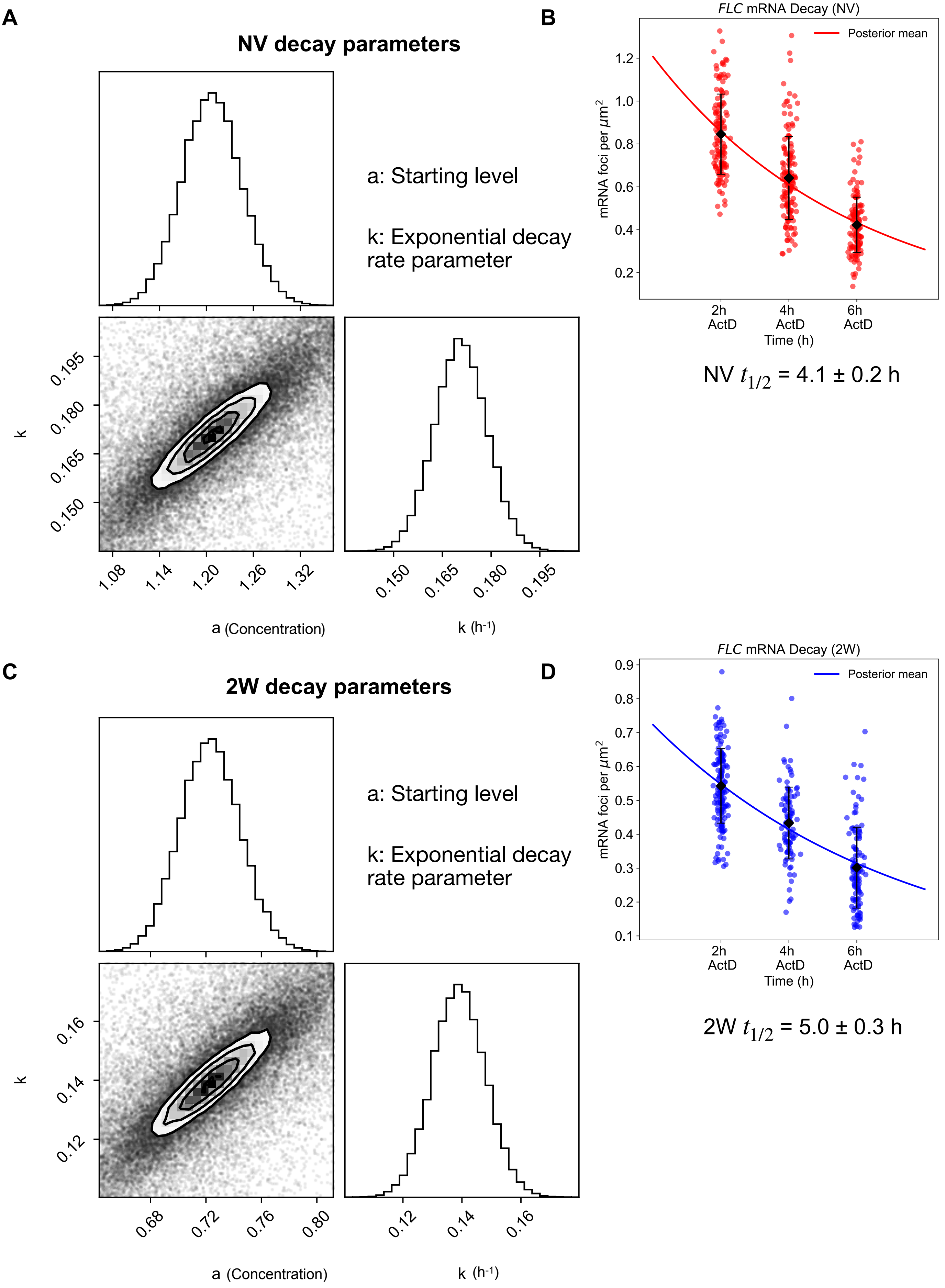

Figure S13. Estimated posterior distribution of the inferred parameters for *FLC* mRNA decay, obtained using a nested sampling approach, for the RNA decay data presented in Figure. 2. (A,C) Individual contour plots show two dimensional projections of the final set of parameter samples generated by the nested sampling algorithm. The histograms show the posterior probability of each inferred parameter (A: NV decay, C: 2W decay). (B,D) Decay curves corresponding to the posterior mean of the parameters, compared to the data (B: NV decay, D: 2W decay). Inferred half-life shown as mean +/- standard deviation of the posterior distribution. For both NV and 2W fits, uniform priors were assumed for both parameters, within the following bounds: 0<a<2; 0<k<1.

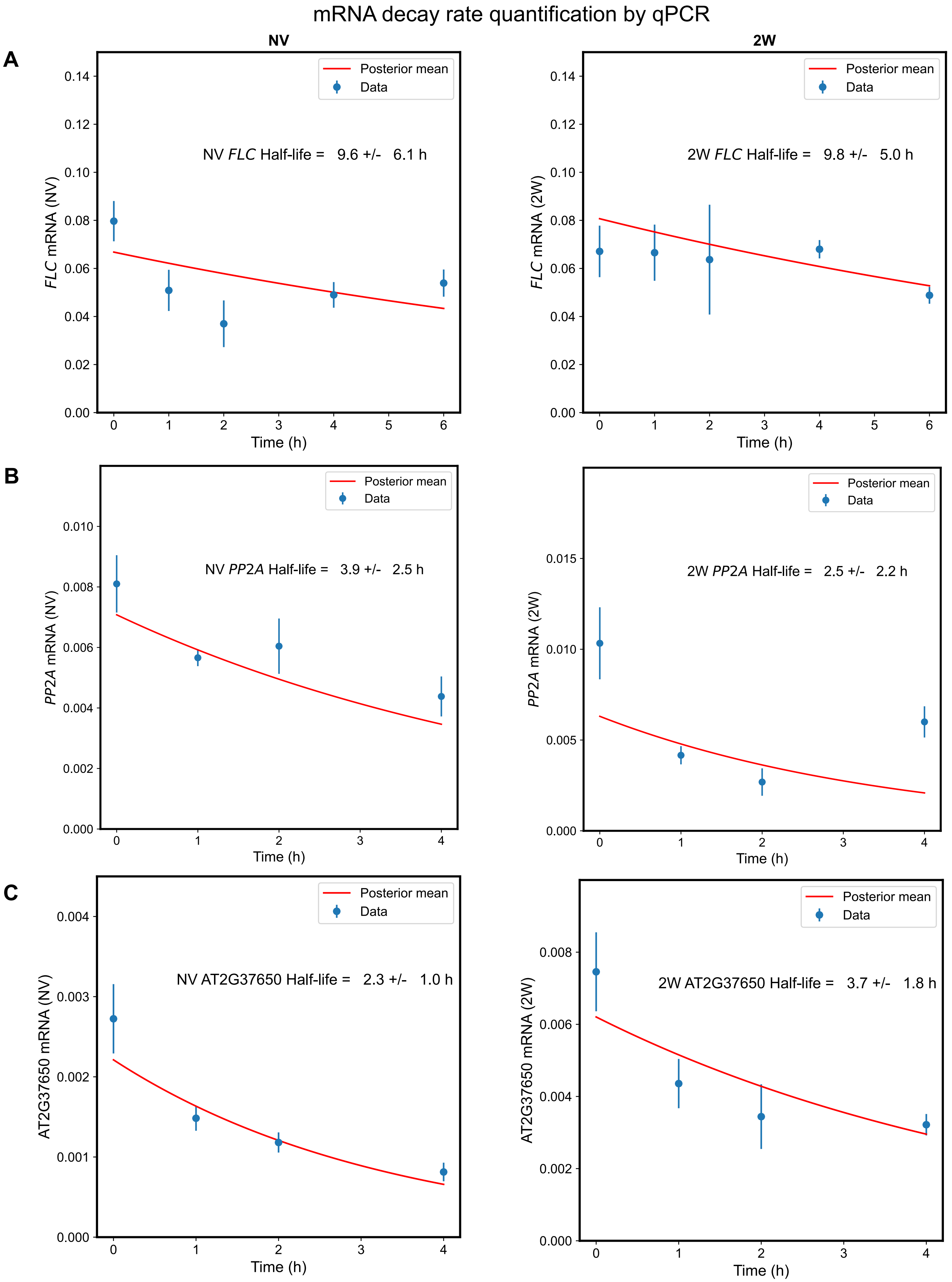

Figure S14. Estimation of RNA decay rates by qPCR following Actinomycin D treatment (see Methods for details). Decay rates were estimated for both non-vernalised (NV) and cold treated (2 weeks) conditions. In all cases, a simple exponential decay rate parameter and starting level was inferred using the nested sampling approach. (A) Measured decay of *FLC* mRNA shown after normalisation by the estimated correction factor (see Methods). The solid line shows the predicted decay corresponding to the mean value of the posterior distribution of the decay rate parameter and the starting level. Half-life estimates are shown as mean +/- standard deviation computed using the mean and standard deviation of the posterior distribution of the decay rate parameter. (B) Measured decay and model prediction for *PP2A* mRNA. (C) Measured decay and model prediction for *AT2G37650* mRNA.

Exponential decay model with non-zero asymptote

A

$$y(t) = a + be^{-kt}$$

B

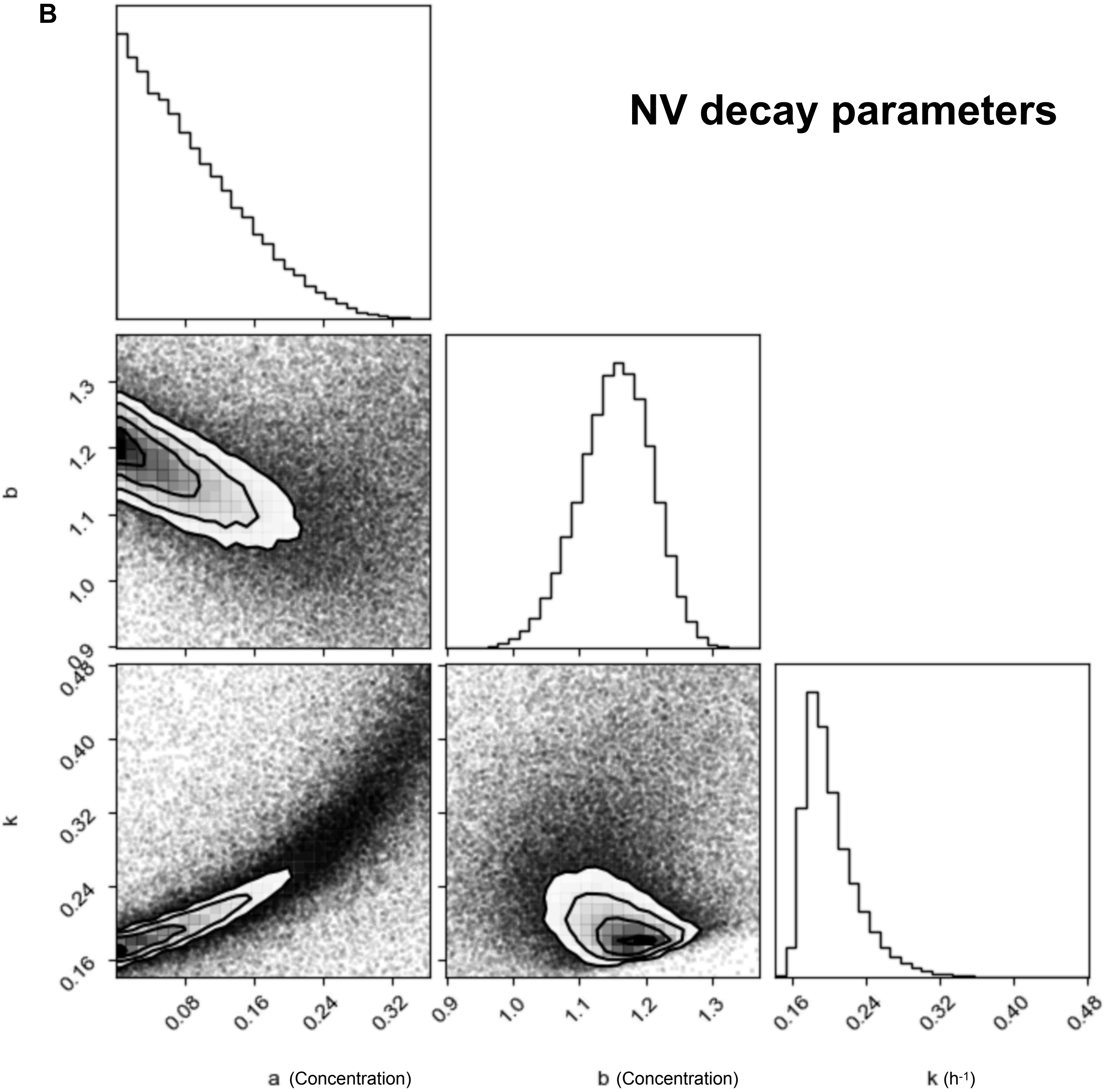

Figure S15. Estimated posterior distribution of the inferred parameters for NV data, obtained using a nested sampling approach, for the RNA decay data presented in Figure 2. (A) Exponential decay model with non-zero asymptote. (B) Individual contour plots show two dimensional projections of the final set of parameter samples generated by the nested sampling algorithm. The histograms show the posterior probability of each inferred parameter. Uniform priors were assumed for all parameters, within the following bounds: $0<a<0.5$; $0<b<2$; $0<k<1$.

Exponential decay model with non-zero asymptote

A

$$y(t) = a + be^{-kt}$$

B

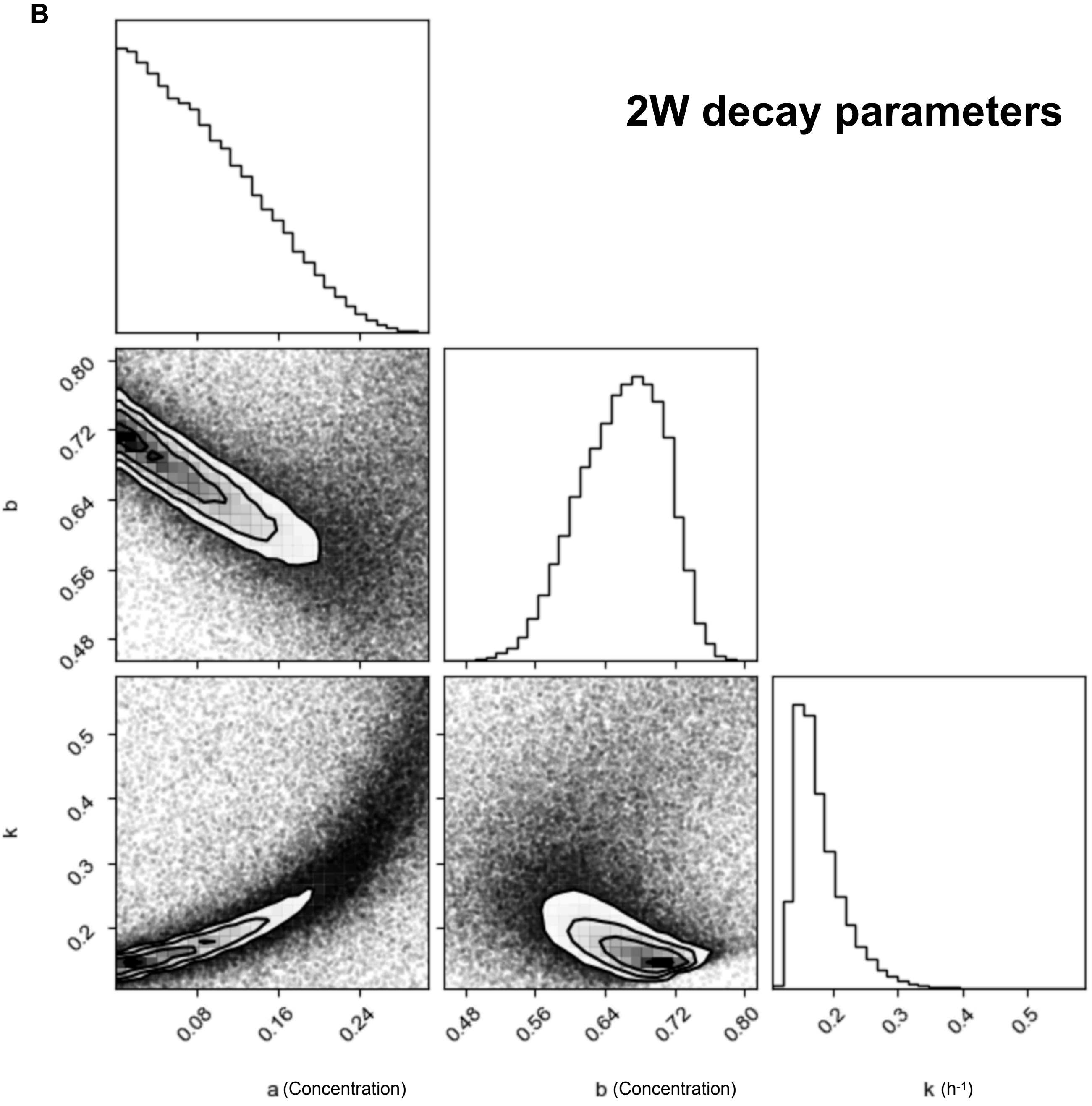

Figure S16. Estimated posterior distribution of the inferred parameters for 2W data, obtained using a nested sampling approach, for the RNA decay data presented in Figure 2. (A) Exponential decay model with non-zero asymptote. (B) Individual contour plots show two dimensional projections of the final set of parameter samples generated by the nested sampling algorithm. The histograms show the posterior probability of each inferred parameter. Uniform priors were assumed for all parameters, within the following bounds: $0<a<0.5$; $0<b<2$; $0<k<1$.

ODE model schematics

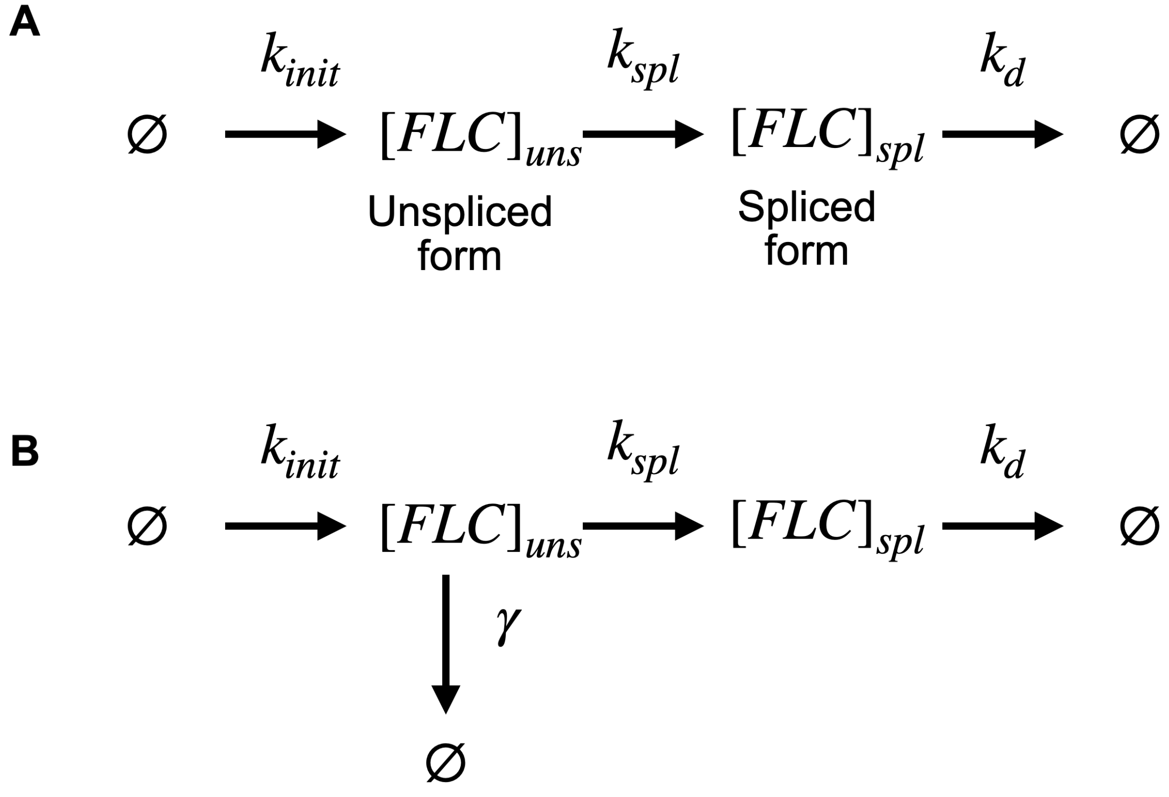

Figure S17. Schematics of ODE models used to analyse differences between spliced and unspliced *FLC* under cold treatment. (A, B) k_{init} represents the transcription initiation rate, k_{spl} represents the splicing rate parameter, k_d represents the degradation rate parameter of the spliced form. (B) γ represents the degradation rate parameter of the unspliced form.

Table S1

Parameter values for *FLC* intron 1 processing model

Timepoints

	Transcription initiation rate	Pol II speed	Intron processing rate	5’-3’ degradation rate	f_{lariat}
**NV**	-	0.04kb/s	0.002/s	0.003kb/s	Fitted
**2WT0**	Fold change inferred	Fold change 0.8	Fold change inferred	0.003kb/s	Fitted
**4WT0**	Fold change inferred	Fold change 0.65	Fold change inferred	0.003kb/s	0.17
**6WT0**	Fold change inferred	Fold change 0.6	Fold change inferred	0.003kb/s	0.17
**8WT0**	Fold change inferred	Fold change 0.6	Fold change inferred	0.003kb/s	0.17
**2WT7**	Fold change inferred	Fold change 0.6	Fold change inferred	0.003kb/s	0.17
**4WT7**	Fold change inferred	Fold change 0.5	Fold change inferred	0.003kb/s	0.17
**6WT7**	Fold change inferred	Fold change 0.3	Fold change inferred	0.003kb/s	0.17
**8WT7**	Fold change inferred	Fold change 0.3	Fold change inferred	0.003kb/s	0.17

Table S1. Parameter table for the *FLC* intron 1 processing model, showing values used for fixed parameters and indicating which parameters were inferred at each timepoint.

Table S2

qPCR primers for mRNA decay experiment

AT5G40370_slow_F	AGGTCTCGCAGAATGGACAG
AT5G40370_slow_R	TCAGTTAACAGCGGAACCAA
AT2G37650_fast_F	GCTAGGGTGATCAGATTGTTTG
AT2G37650_fast_R	GCAACATTGGTTCTCGTGTG
FLC unspliced_F	CGCAATTTTCATAGCCCTTG
FLC unspliced_R	CTTTGTAATCAAAGGTGGAGAGC
FLC spliced F	AGCCAAGAAGACCGAACTCA
FLC spliced R	TTTGTCCAGCAGGTGACATC
PP2A_qPCR_F2	ACTGCATCTAAAGACAGAGTTCC
PP2A_qPCR_R2	CCAAGCATGGCCGTATCATGT

Table S2. Primers used for qPCR analysis of mRNA decay after ActD treatment.
